# An integrated bioinformatics analysis to identify the shared biomarkers in patients with obstructive sleep apnea syndrome and nonalcoholic fatty liver disease

**DOI:** 10.3389/fgene.2024.1356105

**Published:** 2024-07-16

**Authors:** Rou Zhang, Zhijuan Liu, Ran Li, Xiaona Wang, Li Ai, Yongxia Li

**Affiliations:** ^1^ Kunming Medical University, Kunming, China; ^2^ Department of Respiratory Medicine and Critical Care Medicine, The Second Affiliated Hospital of Kunming Medical University, Kunming, China

**Keywords:** OSA, obstructive sleep apnea, nonalcoholic fatty liver disease, Gene Set Enrichment Analysis, receiver operating characteristic, differentially expressed genes

## Abstract

**Background:**

Obstructive sleep apnea (OSA) syndrome and nonalcoholic fatty liver disease (NAFLD) have been shown to have a close association in previous studies, but their pathogeneses are unclear. This study explores the molecular mechanisms associated with the pathogenesis of OSA and NAFLD and identifies key predictive genes.

**Methods:**

Using the Gene Expression Omnibus (GEO) database, we obtained gene expression profiles GSE38792 for OSA and GSE89632 for NAFLD and related clinical characteristics. Mitochondrial unfolded protein response-related genes (UPRmtRGs) were acquired by collating and collecting UPRmtRGs from the GeneCards database and relevant literature from PubMed. The differentially expressed genes (DEGs) associated with OSA and NAFLD were identified using differential expression analysis. Gene Set Enrichment Analysis (GSEA) was conducted for signaling pathway enrichment analysis of related disease genes. Based on the STRING database, protein–protein interaction (PPI) analysis was performed on differentially co-expressed genes (Co-DEGs), and the Cytoscape software (version 3.9.1) was used to visualize the PPI network model. In addition, the GeneMANIA website was used to predict and construct the functional similar genes of the selected Co-DEGs. Key predictor genes were analyzed using the receiver operating characteristic (ROC) curve.

**Results:**

The intersection of differentially expressed genes shared between OSA and NAFLD-related gene expression profiles with UPRmtRGs yielded four Co-DEGs: *ASS1*, *HDAC2*, *SIRT3*, and *VEGFA*. GSEA obtained the relevant enrichment signaling pathways for OSA and NAFLD. PPI network results showed that all four Co-DEGs interacted (except for *ASS1* and *HDAC2*). Ultimately, key predictor genes were selected in the ROC curve, including *HDAC2* (OSA: AUC = 0.812; NAFLD: AUC = 0.729), *SIRT3* (OSA: AUC = 0.775; NAFLD: AUC = 0.750), and *VEGFA* (OSA: AUC = 0.812; NAFLD: AUC = 0.861) (they have a high degree of accuracy in predicting whether a subject will develop two diseases).

**Conclusion:**

In this study, four co-expression differential genes for OSA and NAFLD were obtained, and they can predict the occurrence of both diseases. Transcriptional mechanisms involved in OSA and NAFLD interactions may be better understood by exploring these key genes. Simultaneously, this study provides potential diagnostic and therapeutic markers for patients with OSA and NAFLD.

## Introduction

Obstructive sleep apnea (OSA) is a multifaceted clinical condition with diverse underlying causes that can lead to systemic dysfunction, culminating in complications such as cardiovascular disease, type 2 diabetes, metabolic syndrome, and cognitive impairment. OSA, a prevalent sleep disorder, is characterized by intermittent hypoxemia, hypercapnia, inflammatory responses, oxidative stress, and disrupted sleep patterns (Durán et al., 2001). The escalating prevalence of OSA is attributed to the expanding global economy and population growth. OSA affects approximately 1%–5% of children (Bin-Hasan et al., 2018) and 10%–26% of adults (Sarkar et al., 2018), significantly impacting the overall population health. Effective treatment options for OSA in adults are currently limited, with continuous positive airway pressure therapy being the primary intervention. However, this treatment method exhibits restricted efficacy in addressing metabolic and cardiovascular complications associated with OSA (4). Hence, studying the molecular mechanisms of OSA on related complications and treatments holds considerable clinical importance. Nonalcoholic fatty liver disease (NAFLD) encompasses liver tissue lesions unrelated to alcohol and other specified liver injury factors, including simple hepatic steatosis (NAFL) and nonalcoholic steatohepatitis (NASH), which may progress to cirrhosis and hepatocellular carcinoma (HCC) (Stefan et al., 2019). NAFLD represents a complex metabolic disorder induced by various metabolic abnormalities like obesity, hypertension, hypertriglyceridemia, and diabetes, closely linked with NAFLD development (Younossi et al., 2016). The global prevalence of NAFLD in adults is estimated at 25% (Global burden of 369 diseases, 2020), surpassing hepatitis B as the most widespread cause of chronic liver disease in China, thus imposing a substantial burden on social and health services (Zhou et al., 2020).

OSA and NAFLD are both metabolism-related disorders, each belonging to distinct systems. However, a growing body of research has highlighted the strong relationship and interaction mechanisms between these two conditions. The correlation between OSA and NAFLD has emerged as a significant area of study. The primary pathophysiological feature of OSA is intermittent hypoxia (IH), which can result in damage to various target organs. The liver, being a key metabolic organ, is inevitably affected. This impact is evident not only through abnormal liver enzymes, blood lipids, and glucose metabolism but also through observable changes in liver pathology. Studies indicate that IH can induce liver injury through an excessive inflammatory response, exacerbation of oxidative stress, and impairment of mitochondrial function (Mesarwi et al., 2019). NAFLD, a metabolically demanding liver condition, is closely linked to genetic factors and insulin resistance (IR). Obesity, age, type 2 diabetes, hyperlipidemia, and hypertension are recognized as the five primary risk factors contributing to NAFLD development (Clark and Diehl, 2003; Marchesini et al., 2003). These risk factors are also significantly associated with OSA. Additionally, data from a population-based study suggest that patients with OSA have a higher prevalence of liver diseases, including cirrhosis and NAFLD, when compared to non-OSA individuals (Chou et al., 2015). Animal model studies have revealed that obese mice exposed to intermittent hypoxia exhibit elevated levels of serological markers, liver enzymes, and insulin. Furthermore, liver tissue analysis showed steatosis and lobular inflammation (Drager et al., 2011). Currently, the mechanisms through which OSA influences NAFLD involve factors such as intermittent hypoxia, insulin resistance, oxidative stress, metabolic dysregulation, and intestinal barrier dysfunction. The interplay between OSA and NAFLD, while not entirely elucidated, underscores the intricate relationship between these metabolic disorders. Notably, mitochondrial dysfunction plays a key role in the pathogenesis and progression of metabolic diseases, including OSA and NAFLD. The mitochondrial unfolded protein response (UPRmt) acts as a stress response mechanism triggered by the accumulation of misfolded proteins in the mitochondrial matrix (Tran and Van Aken, 2020a). This response, crucial for proper protein import into the mitochondria, is regarded as a pivotal safeguard for maintaining mitochondrial function (Haynes et al., 2013). Potential key genes involved in the UPRmt have been identified as significant contributors to the pathogenesis of OSA and NAFLD. However, the specific genes implicated in this process remain unidentified. Therefore, leveraging bioinformatics tools for genetic analysis at the molecular level is imperative in deciphering the genetic underpinnings of both disorders.

Traditional biological research often faces challenges in identifying genes and their interactions within databases. With the advancements in sequencing technology and bioinformatics, researchers can now employ bioinformatics analysis to more efficiently pinpoint specific genes associated with certain traits and identify common genes of significant biological importance. This enables the investigation of the correlation and mechanisms of action between diseases. By conducting a thorough bioinformatics analysis, differentially expressed genes (DEGs) between OSA and NAFLD samples were identified. These DEGs were then used to screen differentially co-expressed genes (Co-DEGs) and explore their underlying molecular mechanisms. This study aimed to delve into the related pathways and interaction networks of these genes, ultimately identifying key predictive genes for OSA and NAFLD.

## Materials and methods

**FIGURE 1:**
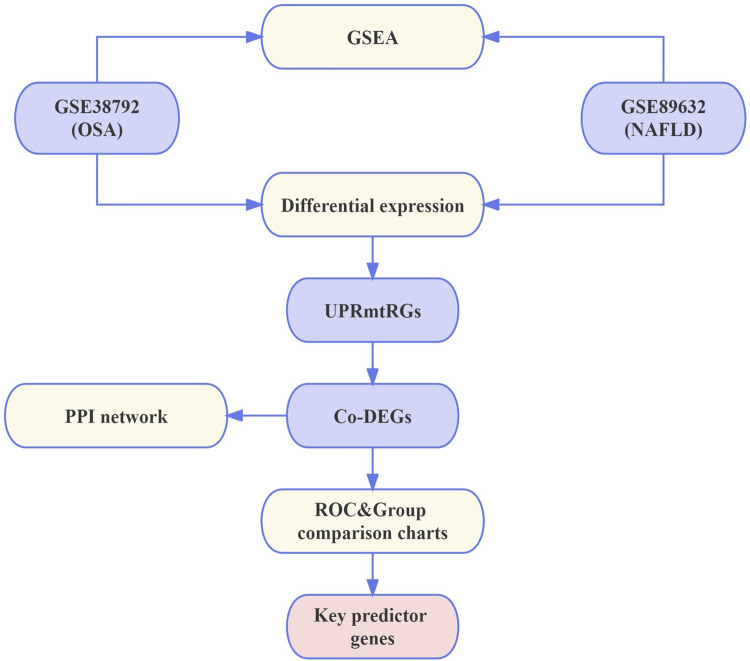
Flowchart. NAFLD, nonalcoholic fatty liver disease; OSA, obstructive sleep apnea; UPRmtRGs, mitochondrial unfolded protein response-related genes; GSEA, Gene Set Enrichment Analysis; Co-DEGs, differentially co-expressed genes; PPI, protein–protein interaction; ROC, receiver operating characteristic.

The workflow detailing the analysis and extraction of key predictive genes is illustrated in Figure 1.

### Data preparation

Gene expression profiles and related clinical data from two datasets were obtained for OSA (GSE38792) (Gharib et al., 2013) and NAFLD (GSE89632) (Arendt et al., 2015a) using GEOquery of the R package (Davis and Meltzer, 2007) from the GEO database (Barrett et al., 2013). The GSE38792 dataset, sourced from *Homo sapiens*, utilized the GPL6244 [HuGene-1_0-st] Affymetrix Human Gene 1.0 ST Array [transcript (gene) version]. It included microarray gene expression profiles from 10 OSA patient samples (case group) and 8 normal visceral adipose tissue samples (normal group). The GSE89632 dataset from *Homo sapiens* was based on the expression bead chip GPL14951 Illumina HumanHT-12 WG-DASL V4.0 R2, containing data from 39 NAFLD patient samples (case group) and 24 normal liver samples (normal group). All samples were included in the study, and the dataset probe naming used the corresponding GPL-covered platform file. Specific dataset information is given in Table 1. The GeneCards database (Stelzer et al., 2016) offers comprehensive gene information about humans. Mitochondrial unfolded protein response-related genes (UPRmtRGs) were collected from the GeneCards database using the search term “mitochondrial unfolded protein response” with a relevance score threshold >0.2, resulting in a total of 78 UPRmtRGs. Additional UPRmtRGs were identified through a PubMed literature search (Ge et al., 2019; Tran and Van Aken, 2020), totaling 32 genes. Merging the UPRmtRGs from both sources yielded 102 antioxidant-related genes (ARGs) for subsequent analysis. Specific gene names are given in Supplementary Table S1.

**TABLE 1:** List of dataset information.

Items	GSE38792	GSE89632
Platform	GPL6244	GPL14951
Species	*Homo sapiens*	*Homo sapiens*
Disease	OSA	NAFLD
Tissue	Visceral adipose tissue	Liver
Samples in the case group	39	10
Samples in the normal group	24	8
Reference	Sarkar et al. (2018)	Light et al. (2018)

OSA, obstructive sleep apnea; NAFLD, nonalcoholic fatty liver disease.

### Differential expression analysis

To investigate the biological mechanisms, traits, and pathways of target genes in individuals with OSA and NAFLD, we initially used the limma plug-in within R 4.1.2 software (Ritchie et al., 2015) to standardize the datasets GSE38792 and GSE89632. These datasets were then divided into disease and normal groups, and the processed expression profile data were analyzed. Through this process, DEGs were identified within the various OSA and NAFLD dataset groups, with upregulated genes defined as those with logFC >0.1 and *p* < 0.05 and downregulated genes as those with logFC < −0.1 and *p* < 0.05. The differential analysis outcomes were visualized using volcano plots created using the ggplot2 plug-in in R 4.1.2 software. Subsequently, we compared the identified OSA and NAFLD differentially expressed genes with the UPRmtRGs to pinpoint Co-DEGs, which were illustrated using Venn diagrams. Furthermore, the expression patterns of Co-DEGs in datasets GSE38792 and GSE89632 were displayed as a heatmap generated using R 4.1.2 software.

### Gene Set Enrichment Analysis

To determine the contribution of each gene to the phenotype, Gene Set Enrichment Analysis (GSEA) was utilized (Subramanian et al., 2005). GSEA evaluates the distribution trend of a specified gene set in a gene table sorted based on the phenotypic correlation. In this study, the clusterProfiler plug-in in R 4.1.2 software was employed to enrich all genes in the disease and normal groups of datasets GSE38792 and GSE89632. The GSEA parameters included a 2021 seed, 10,000 computation times, each gene set containing a minimum of 5 genes, a maximum of 500 genes per set, and the utilization of the Benjamini–Hochberg (BH) method for *p*-value correction. The gene set “c2.cp.v7.2.symbols” was obtained from the Molecular Signatures Database (MSigDB). Substantial enrichment was determined based on the false discovery rate (FDR) value (q-value) < 0.25 and *p*-value <0.05.

### Protein–protein interaction network construction and module analysis

We utilized the STRING database to construct a protein–protein interaction (PPI) network associated with Co-DEGs. The STRING database is a valuable resource that identifies known proteins and predicts their interactions (Szklarczyk et al., 2019). A minimum interaction score of 0.150, signifying medium confidence, was set for the inclusion of interactions in the network. In PPI networks, densely interconnected local clusters may represent specific chemical complexes with distinct biological functions. Furthermore, the maximal clique centrality (MCC) algorithm, a well-established metric in bioinformatics, was employed to assess network performance. The PPI network model was visualized using Cytoscape software (version 3.9.1) (Shannon et al., 2003). To predict functionally related genes among the screened Co-DEGs, the GeneMANIA website (Franz et al., 2018) was utilized. Following the Co-DEG screening, functional gene predictions were made using GeneMANIA, subsequently establishing an interaction network.

### Statistical analysis

All data processing and analysis in this study were conducted using R software version 4.1.2. Continuous variables were presented as the mean ± standard deviation. In comparison of two sets of continuous variables, the statistical significance of normally distributed variables was assessed using the Wilcoxon rank sum test or independent Student’s *t*-test. For comparison of categorical variables, the chi-square test or Fisher’s exact test was used. The receiver operating characteristic (ROC) curve (Mandrekar, 2010) was generated using the pROC package in R 4.1.2. Spearman’s correlation analysis was utilized to determine the correlation coefficient between different molecules in the absence of specific instructions. A *p*-value less than 0.05 was considered statistically significant.

## Results

### Identification of DEGs

**FIGURE 2:**
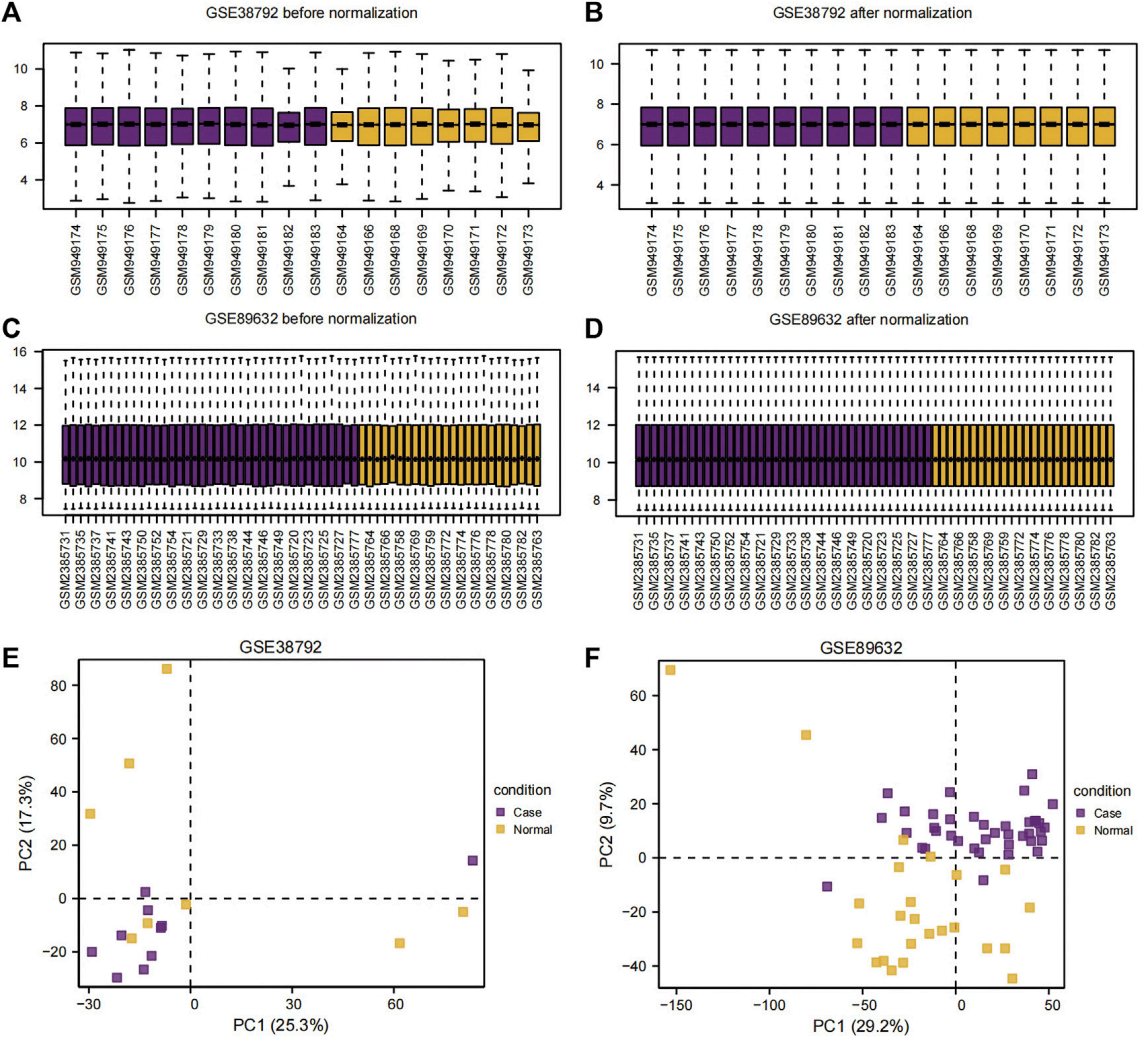
Sample distribution box plot and PCA plot. **(A,B)** Sample distribution box plot before **(A)** and after **(B)** the dataset GSE38792 merged. **(C,D)** Sample distribution box plots before **(C)** and after **(D)** the dataset GSE89632 merged. **(E,F)** PCA plots of the datasets GSE38792 **(E)** and GSE89632 **(F)**. PCA, principal component analysis.

**FIGURE 3:**
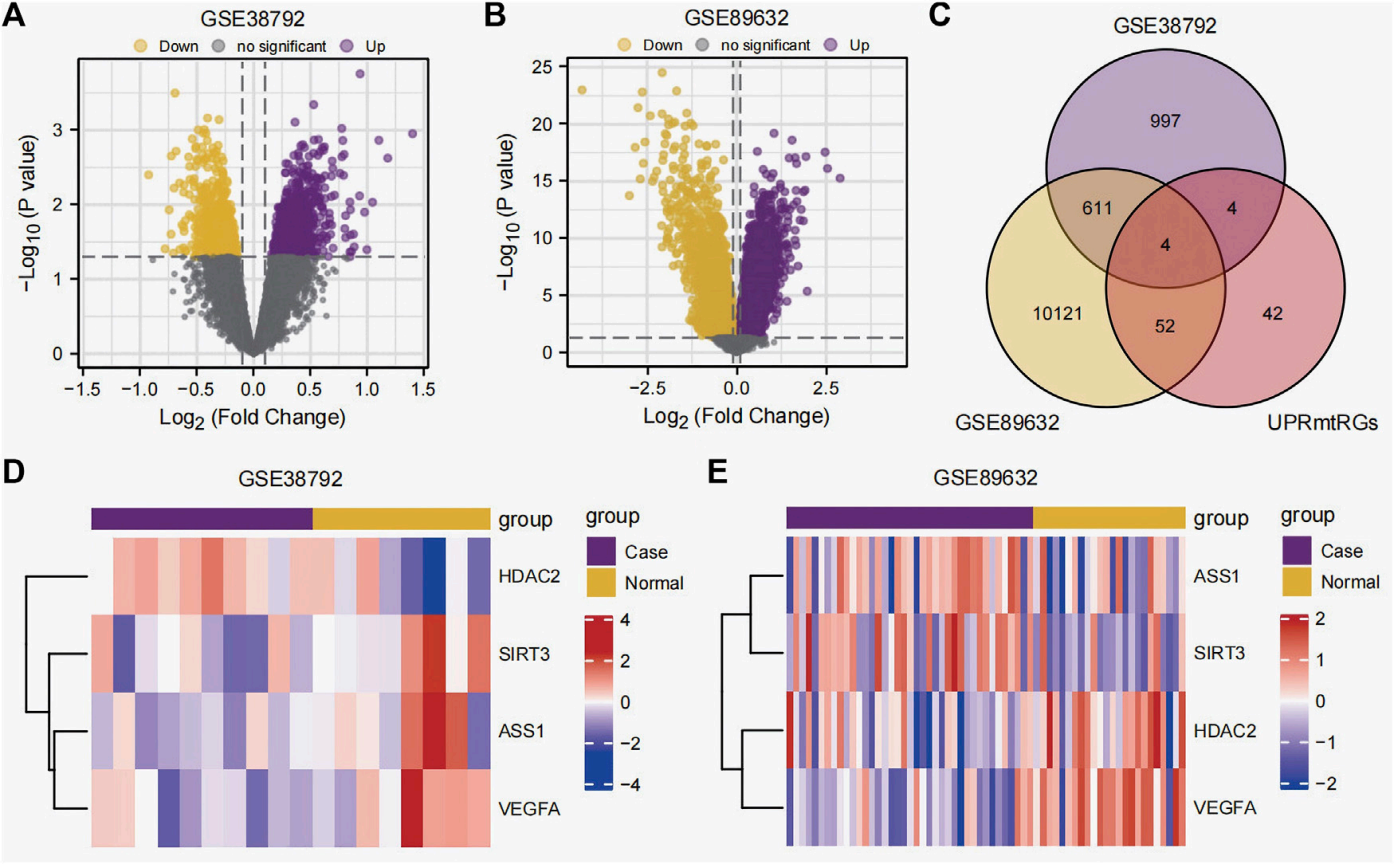
Differential expression analysis. **(A,B)** Volcano plot of differential expression analysis of the datasets GSE38792 **(A)** and GSE89632 **(B)** between the disease (group: case) and normal (group: normal). **(C)** Venn diagram of the intersection of differentially expressed genes and UPRmtRGs obtained from datasets GSE38792 and GSE89632. **(D,E)** Heatmap of the expression levels of Co-DEGs in the datasets GSE38792 and GSE89632. UPRmtRGs, mitochondrial unfolded protein response-related genes; Co-DEGs, differentially co-expressed genes.

The datasets GSE38792 and GSE89632 were normalized and compared before and after standardization through distribution box plot analysis (Figures 2A–D) and principal component analysis (PCA) plots (Figures 2E, F). The results indicated that post-standardization, the expression patterns of samples in both GSE38792 and GSE89632 became more consistent, demonstrating good intra-group reproducibility and intergroup differentiation between the disease and normal groups. A total of 21,408 differentially expressed genes were identified in dataset GSE38792, meeting the criteria of *p* < 0.05 and |logFC| > 0.1. Among these, 851 genes were found to have high expression in the OSA high-risk group (considered upregulated genes), while 765 genes exhibited low expression in the same group. This differential gene expression analysis was visualized through volcano plots (Figure 3A). In dataset GSE89632, 20,819 differentially expressed genes were discovered, with 10,778 genes meeting the thresholds of |logFC| > 0.1 and *p* < 0.05. In this dataset, 5,947 genes were upregulated and 4,841 genes were downregulated in the NAFLD high-risk group compared to the low-risk group. A visualization of these results was presented in volcano plots (Figure 3B). Subsequently, an intersection analysis was carried out among the differentially expressed genes in OSA, NAFLD, and UPRmtRG datasets, revealing four common genes (*ASS1*, *HDAC2*, *SIRT3*, and *VEGFA*), which were illustrated using a Venn diagram (Figure 3C). Lastly, a heatmap was generated (Figures 3D, E) to display the expression levels of these four Co-DEGs in datasets GSE38792 and GSE89632.

### Gene Set Enrichment Analysis

**FIGURE 4:**
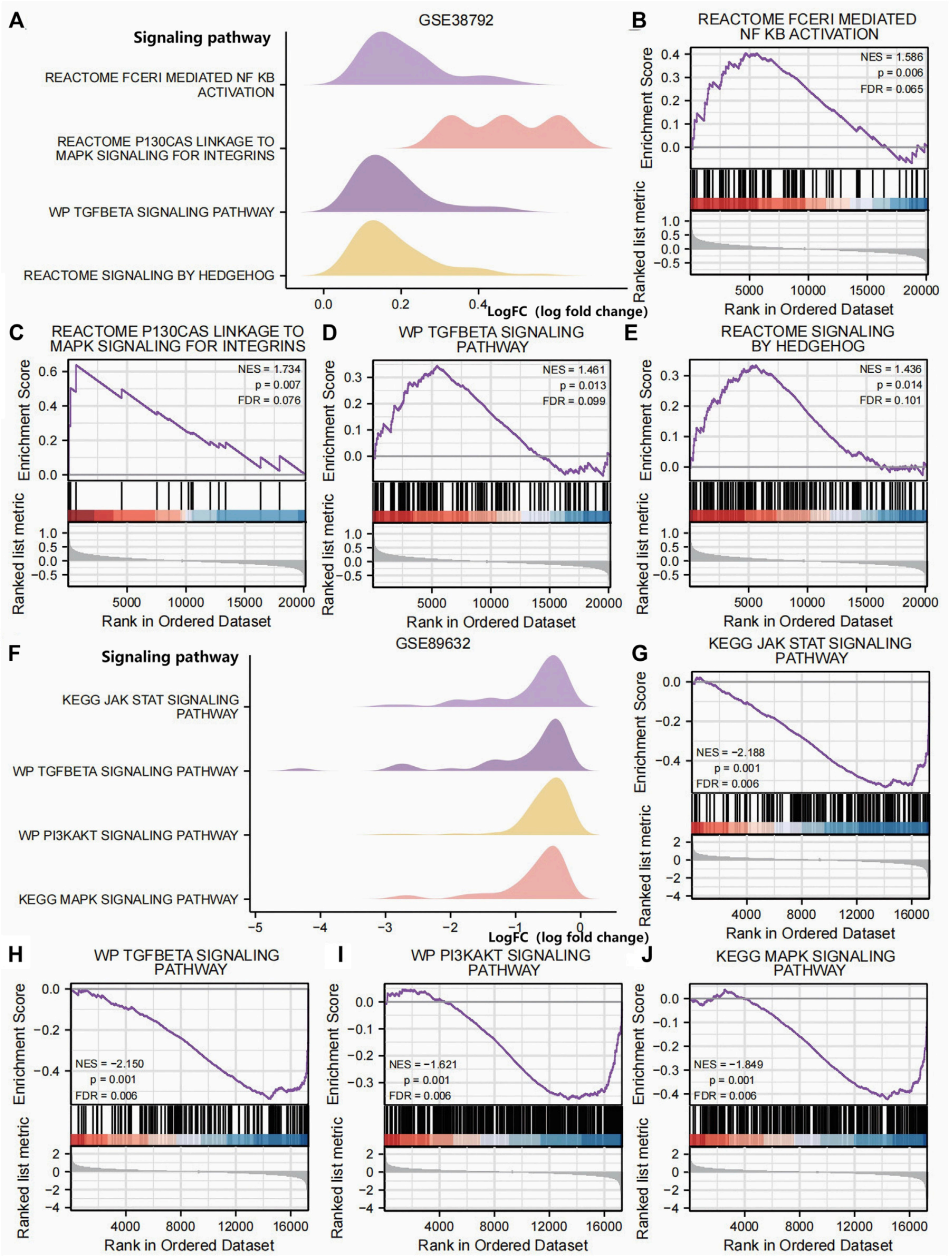
GSEA **(A)**. Ridge plot of the GSEA of dataset GSE38792; GSEA of dataset GSE38792 yielded mainly four biological features. **(B–E)** Pathway maps of the GSEA of dataset GSE38792. Genes in dataset GSE38792 showed significant enrichment in pathways such as REACTOME FCERI-MEDIATED NF KB ACTIVATION **(B)**, REACTOME P130 CAS LINKAGE TO MAPK SIGNALING FOR INTEGRINS **(C)**, WP TGF BETA SIGNALING PATHWAY **(D)**, and REACTOME SIGNALING BY HEDGEHOG **(E)**. **(F)** Ridge plot of the GSEA of dataset GSE89632. The GSEA of dataset GSE89632 yielded mainly four biological features. **(G–J)** Pathway maps of the GSEA of dataset GSE89632. Genes in the dataset GSE89632 showed significant enrichment in pathways such as KEGG JAK STAT SIGNALING PATHWAY **(G)**, WP TGF BETA SIGNALING PATHWAY **(H)**, WP PI3KAKT SIGNALING PATHWAY **(I)**, and KEGG MAPK SIGNALING PATHWAY **(J)**. *p* < 0.05 and an FDR value (q-value) < 0.25 were the significant enrichment screening parameters for the GSEA. GSEA, Gene Set Enrichment Analysis.

In order to determine the impact of gene expression levels on the difference between the disease group and the normal group, we analyzed the relationship between gene expression, biological processes, cellular components affected, and molecular functions involved in datasets GSE38792 and GSE89632 using GSEA. We applied stringent enrichment screening criteria, defining significance as *p* < 0.05 and FDR value (q-value) <0.25. Our results revealed a notable enrichment of genes in dataset GSE38792 in pathways such as REACTOME FCERI-MEDIATED NF KB ACTIVATION, REACTOME P130 CAS LINKAGE TO MAPK SIGNALING FOR INTEGRINS, WP TGF BETA SIGNALING PATHWAY, and REACTOME SIGNALING BY HEDGEHOG, as depicted in Figures 4B–E; Table 2. Additionally, we visualized the data through ridge plots and pathway maps in the dataset TCGA-COADREAD (Figures 4A–E). Conversely, genes in dataset GSE89632 exhibited significant enrichment in pathways including KEGG JAK STAT SIGNALING PATHWAY, WP TGF BETA SIGNALING PATHWAY, WP PI3K-AKT SIGNALING PATHWAY, and KEGG MAPK SIGNALING PATHWAY, as shown in Figures 4G–J; Table 3. Similar to the previous dataset, we utilized ridge plots and pathway maps to display the results in the dataset TCGA-COADREAD (Figures 4F–I).

**TABLE 2:** GSEA of dataset GSE38792.

Description	Set size	Enrichment score	NES	*p*-value	q-value
REACTOME CELL CYCLE MITOTIC	453	0.399311539	1.955092057	0.000139218	0.007625044
WP VEGFA VEGFR2 SIGNALING PATHWAY	395	0.318495785	1.542000725	0.000141143	0.007625044
REACTOME CLASS I MHC-MEDIATED ANTIGEN PROCESSING PRESENTATION	333	0.35495257	1.694858866	0.00014374	0.007625044
REACTOME M PHASE	316	0.43204613	2.051897554	0.000145033	0.007625044
REACTOME ASPARAGINE N-LINKED GLYCOSYLATION	266	0.387808776	1.811180513	0.000147689	0.007625044
REACTOME ORGANELLE BIOGENESIS AND MAINTENANCE	250	0.387058632	1.795034965	0.000148368	0.007625044
REACTOME PLATELET ACTIVATION SIGNALING AND AGGREGATION	244	0.345733742	1.59952342	0.00014839	0.007625044
REACTOME DNA REPAIR	251	0.391426592	1.814982473	0.000148633	0.007625044
REACTOME CELL CYCLE CHECKPOINTS	233	0.435028556	2.001351684	0.000149723	0.007625044
REACTOME RHO GTPASE EFFECTORS	238	0.379048484	1.746110214	0.000150083	0.007625044
REACTOME DEUBIQUITINATION	220	0.360639403	1.647550585	0.000150784	0.007625044
REACTOME FCERI-MEDIATED NF KB ACTIVATION	77	0.404494579	1.585842553	0.006012024	0.064962718
REACTOME P130CAS LINKAGE TO MAPK SIGNALING FOR INTEGRINS	15	0.638084798	1.733972301	0.007993909	0.076478233
WP TGFBETA SIGNALING PATHWAY	128	0.344302402	1.46128931	0.013130504	0.09869798
REACTOME SIGNALING BY HEDGEHOG	137	0.335039805	1.435574434	0.013992686	0.101217951

GSEA, Gene Set Enrichment Analysis.

**TABLE 3:** GSEA of dataset GSE89632.

Description	Set size	Enrichment score	NES	P-value	Q values
Reactome gpcr ligand binding	435	−0.345270488	−1.582097984	0.000133476	0.005819216
Reactome signaling by interleukins	427	−0.486109881	−2.223201608	0.000134336	0.005819216
WP vegfa vegfr2 signaling pathway	411	−0.360100378	−1.641413424	0.000135135	0.005819216
KEGG olfactory transduction	381	−0.617695066	−2.797025693	0.000136705	0.005819216
Reactome olfactory signaling pathway	374	−0.607846828	−2.745263814	0.000137438	0.005819216
Naba secreted factors	325	−0.443081706	−1.977235319	0.000139276	0.005819216
Reactome class A 1 rhodopsin like receptors	307	−0.381423481	−1.692225661	0.000141423	0.005819216
Wp nuclear receptors metapathway	307	−0.424678126	−1.884129471	0.000141423	0.005819216
Reactome extracellular matrix organization	286	−0.428167739	−1.886043407	0.000142531	0.005819216
WP IL18 signaling pathway	262	−0.463689704	−2.028176355	0.000143719	0.005819216
Naba core matrisome	255	−0.407800593	−1.77711959	0.000144823	0.005819216
KEGG jak stat signaling pathway	151	−0.534655611	−2.187984929	0.000153468	0.005819216
WP TGFBETA signaling pathway	129	−0.537025337	−2.149688837	0.000157109	0.005819216
WP PI3KAKT signaling pathway	333	−0.362479724	−1.620986548	0.000139005	0.005819216
KEGG mapk signaling pathway	257	−0.423841351	−1.849335982	0.000144363	0.005819216

GSEA, Gene Set Enrichment Analysis.

### The PPI interaction network

**FIGURE 5:**
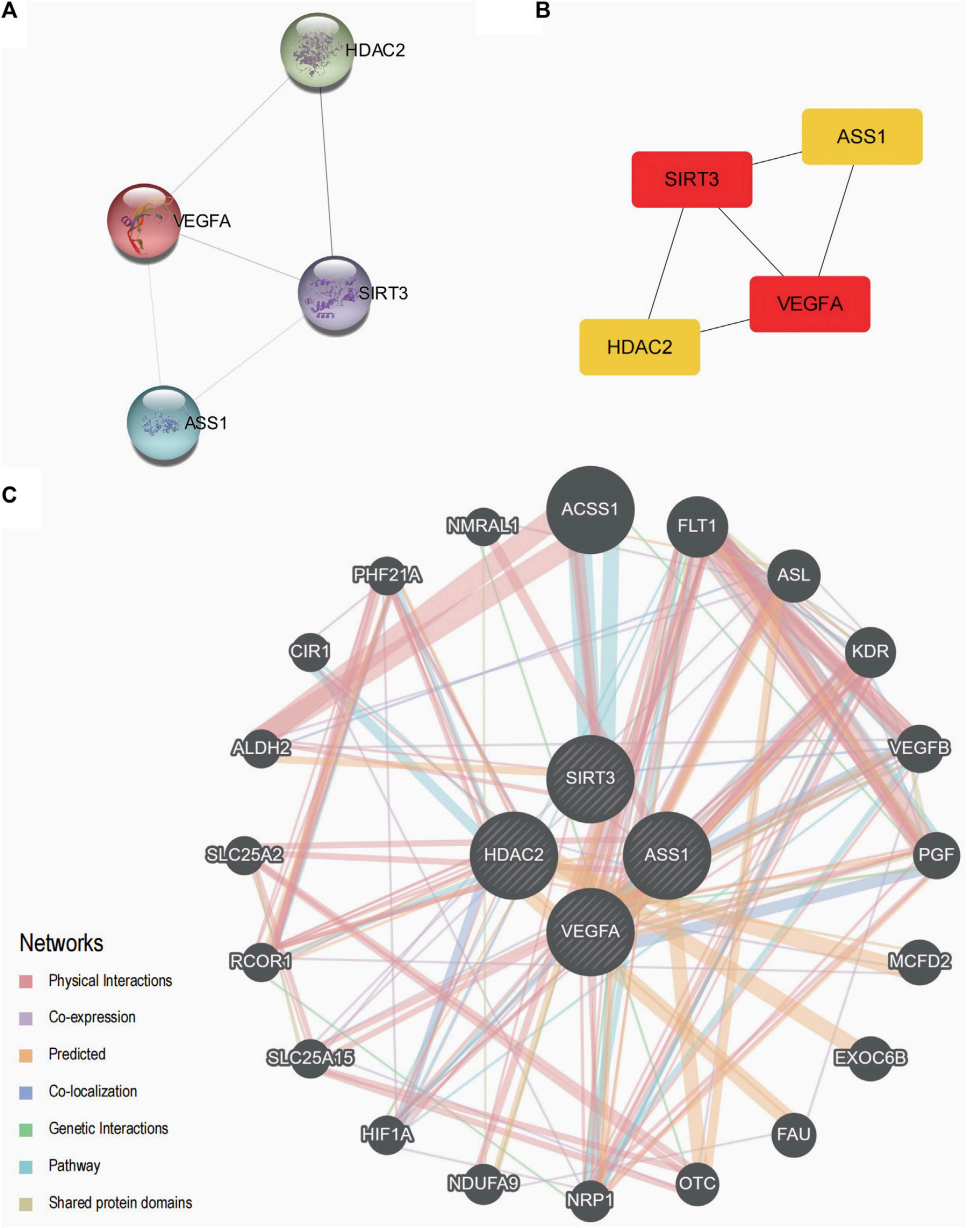
PPI interaction network. **(A)** PPI network of Co-DEGs. **(B)** PPI network of Co-DEGs in the MCC algorithm; the color of the rectangle in the figure from yellow to red represents the gradual increase in the score. **(C)** The GeneMANIA website predicts functionally similar gene interaction networks of Co-DEGs. **(A,B)** Interconstructing networks are collected in the STRING database and built using Cytoscape software, with a minimum interaction score of 0.150. The infrastructure network in **(C)** was collected and exported on the GeneMANIA website. In this diagram, input Co-DEGs are represented by black circles with white slashes, anticipated functionally related genes are represented by other black circles without white slashes, physical interactions between genes are represented by red lines, co-expression correlations between genes are represented by purple lines, predicted relationships between genes are represented by orange lines, co-localization relationships between genes are represented by blue lines, genetic interaction relationships between genes are represented by green lines, pathway connections between genes are represented by pale blue lines, and shared protein domain linkages between genes are represented by yellow–green lines. Co-DEGs, differentially co-expressed genes; PPI, protein–protein interaction; MCC, maximal clique centrality.

We conducted a protein–protein interaction analysis on four Co-DEGs (*ASS1*, *HDAC2*, *SIRT3*, and *VEGFA*) using the STRING database. The minimum required interaction score in the STRING database was set to medium confidence at 0.150. This score was chosen as the threshold to construct the PPI network for the four Co-DEGs. The interaction relationships were visualized using Cytoscape software (see Figure 5A). The results showed that, with a minimum interaction score of 0.150, all Co-DEGs, except *ASS1* and *HDAC2*, exhibited interactions with at least one other Co-DEG. Subsequently, the MCC algorithm was used to calculate the scores of the Co-DEGs associated with the nodes in the PPI network. The Co-DEGs were then ranked based on these scores, visualized using a gradient color scale from red to yellow. As shown in Figure 5B, *SIRT3* and *VEGFA* are tied for the top position in the MCC algorithm score ranking. These four genes were identified to play crucial roles in the PPI network, with *SIRT3* and *VEGFA* receiving the highest scores in the MCC analysis, underscoring their roles as core key genes in the diseases under investigation. Detailed gene scores are given in Supplementary Table S2. Additionally, we utilized the GeneMANIA website to predict and construct a functionally similar gene interaction network for these four Co-DEGs (see Figure 5C), allowing us to explore their physical interactions, co-expression patterns, predictions, colocalization, gene interactions, pathways, shared protein domains, and other relevant information.

### Group comparison charts and the ROC curves

**FIGURE 6:**
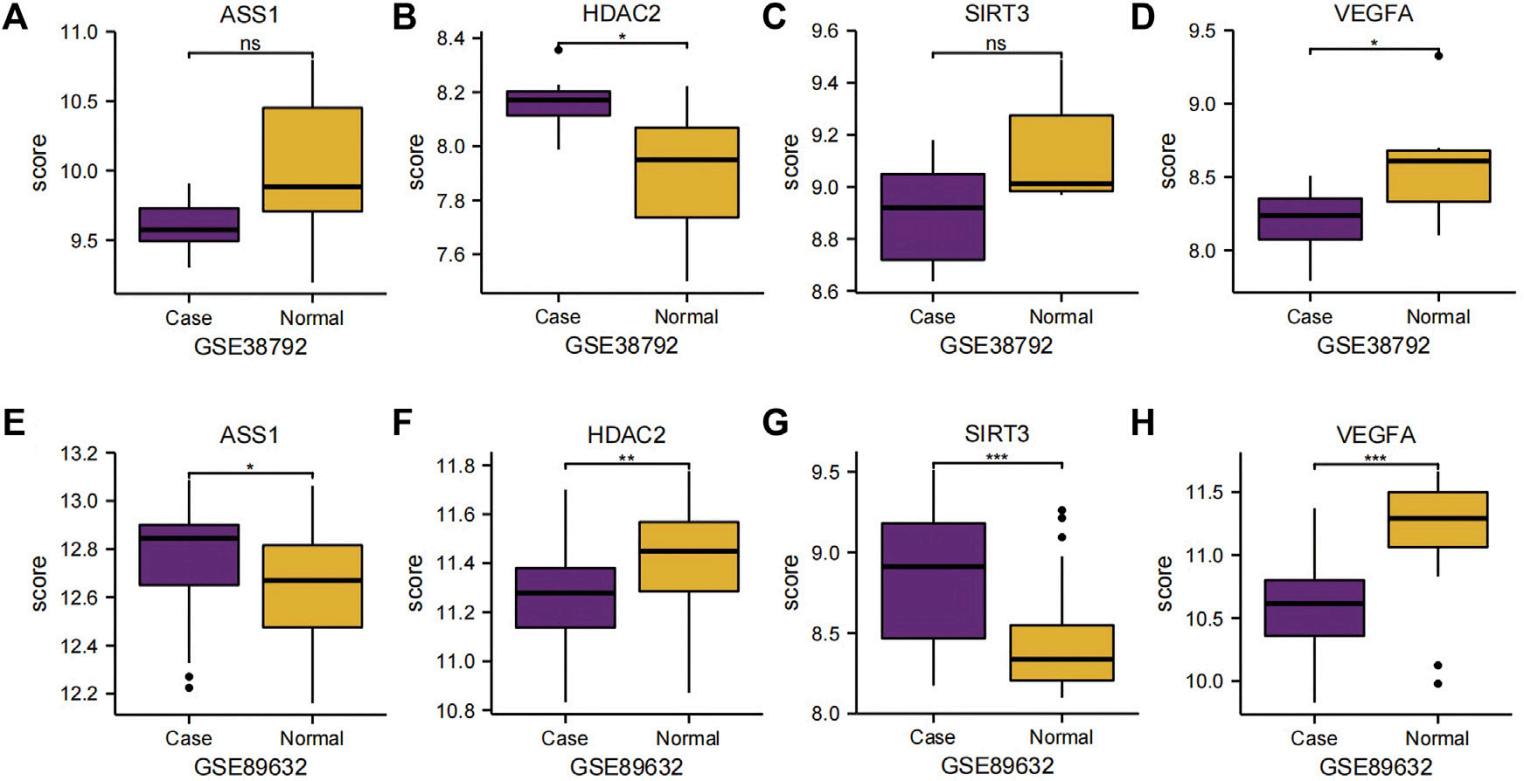
Group comparison charts of Co-DEGs. **(A–D)** Group comparison charts of the expression of genes *ASS1* **(A)**, *HDAC2* **(B)**, *SIRT3* **(C)**, and *VEGFA* **(D)** in dataset GSE38792. **(E–H)** Group comparison charts of the expression of genes *ASS1* **(E)**, *HDAC2* **(F)**, *SIRT3* **(G)**, and *VEGFA* **(H)** in dataset GSE89632. The following symbols represent different levels of statistical significance: * denotes a level of significance equal to *p* < 0.05, is statistically significant; ** denotes a level of significance equal to *p* < 0.01, is highly statistically significant; *** denotes a level of significance equal to *p* < 0.001, is extremely statistically significant; and ns stands for not statistically significant. Co-DEGs, differentially co-expressed genes.

We subsequently analyzed the group comparison charts of the expression levels of the four Co-DEGs (*ASS1*, *HDAC2*, *SIRT3*, and *VEGFA*) in the datasets GSE38792 and GSE89632 (Figures 6A–H). The results showed that there was no statistically significant difference in the expressions of *ASS1* and *SIRT3* (*p* ≥ 0.05), while the expressions of *HDAC2* and *VEGFA* exhibited statistical significance (*p* < 0.05) between the disease group and normal group in dataset GSE38792. Furthermore, a statistically significant difference in the expression of *ASS1* (*p* < 0.05), a highly statistically significant difference in the expression of *HDAC2* (*p* < 0.01), and an extremely statistically significant difference in the expressions of *SIRT3* and *VEGFA* (*p* < 0.001) were observed between the disease group and normal group in dataset GSE89632.

**FIGURE 7:**
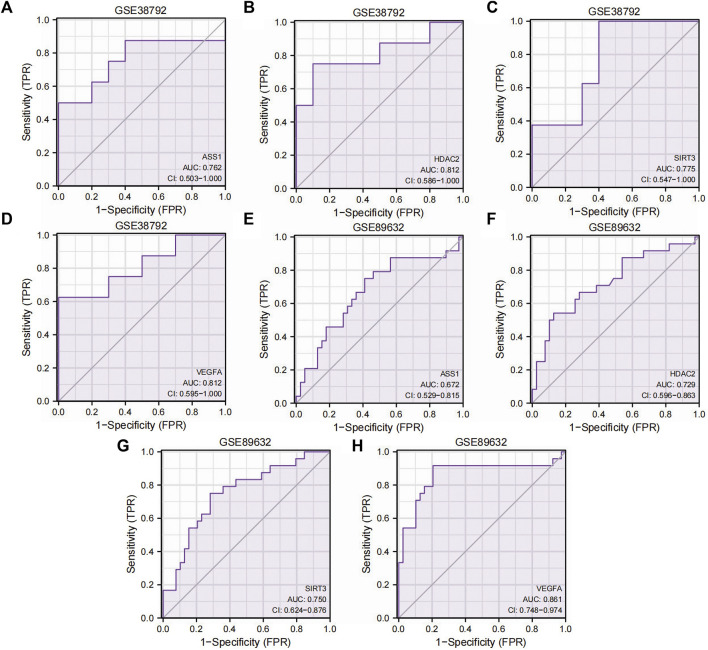
ROC curves of the Co-DEGs. **(A–D)** ROC curve results of genes *ASS1* **(A)**, *HDAC2* **(B)**, *SIRT3* **(C)**, and *VEGFA* **(D)** in dataset GSE38792. **(E–H)** ROC curve results of genes *ASS1* **(E)**, *HDAC2* **(F)**, *SIRT3* **(G)**, and *VEGFA* **(H)** in dataset GSE89632. TPR, true positive rate; FPR, false positive rate; Co-DEGs, differentially co-expressed genes; ROC, receiver operating characteristic; CI, confidence interval.

To investigate the relationship between the expressions of the four Co-DEGs (*ASS1*, *HDAC2*, *SIRT3*, and *VEGFA*) and the incidence of OSA and NAFLD, we generated ROC curves of these genes in datasets GSE38792 and GSE89632 and presented the findings (Figures 7A–H). The ROC curves indicated that in the OSA dataset GSE38792, the AUC values for *ASS1*, *HDAC2*, *SIRT3*, and *VEGFA* are 0.762, 0.812, 0.775, and 0.812, respectively (all above 0.7), suggesting their high accuracy in predicting the correct classification of case and normal groups. In the NAFLD dataset GSE89632, the AUC was 0.672 for *ASS1*, indicating lower accuracy in predicting the correct classification of case and normal groups, whereas the AUC values for *HDAC2*, *SIRT3*, and *VEGFA* are 0.729, 0.750, and 0.861, respectively (over 0.7), signifying their high accuracy in predicting the correct classification of case and normal groups. These significant predictive genes may serve as valuable markers for the potential diagnosis and treatment of OSA and NAFLD.

## Discussion

OSA is a chronic, progressive disease that is widespread in the population, and IH and the resulting oxidative stress response are hallmark manifestations of OSA. In addition, sleep disorders are common in OSA patients, and a large number of studies (Koritala et al., 2021; Soreca, 2021) show that OSA patients may have circadian rhythm disorders, and the two may interact to cause metabolic disorders. NAFLD is also a prevalent metabolic disease in the population and may severely impair liver function, especially after progression to NASH, and the liver disease spectrum may further develop into hepatocellular carcinoma (Ye et al., 2020). Progression of the liver disease spectrum is associated with an increased incidence of metabolic disease, and metabolic-targeted therapies may alleviate the progression of hepatocellular carcinoma and can be used for other malignancies (Gnocchi et al., 2023).

OSA and NAFLD are linked to metabolism, and metabolic disorders have emerged as risk factors for various malignancies (Gnocchi et al., 2023). Studies focusing on both conditions can provide valuable insights into the role of metabolic disorders in disease development. Research findings (Tanné et al., 2005; Drager et al., 2011; Sookoian and Pirola, 2013; Petta et al., 2015; Kang et al., 2017) indicate a close association between OSA and NAFLD, although there is an ongoing debate and a lack of high-quality clinical evidence to definitively establish a causal or concurrent relationship between OSA and NAFLD. Nonetheless, the majority of studies have highlighted the impact of OSA on NAFLD. The recent renaming of NAFLD to metabolism-related fatty liver disease (Eslam et al., 2022) underscores the growing interest in exploring the connection between OSA and NAFLD within the realm of metabolism. Notably, CPAP therapy is currently the preferred treatment for OSA, and studies suggest that CPAP treatment can enhance the liver function in OSA patients with NAFLD (Kim et al., 2018). However, noncompliance with CPAP therapy is common among OSA patients, leading to the progression of OSA and its associated complications. Therefore, it is imperative to identify common predictor genes and investigate the molecular mechanisms underlying the interaction between OSA and NAFLD to facilitate early diagnosis and effective treatment strategies. While the biological system is complex, and genes represent just one aspect of the disease phenotype, genetic research remains pivotal in guiding clinical and fundamental investigations.

In this study, we analyzed datasets on OSA and NAFLD using differential expression analysis and GSEA to identify Co-DEGs and related signal transduction pathways. For the first time, we utilized UPRmtRGs to identify core Co-DEGs by merging DEGs and UPRmtRGs from both diseases, providing key genes for future metabolic disease research. Additionally, we employed ROC analysis to identify crucial predictor genes between OSA and NAFLD. Our bioinformatics analysis revealed four key differential genes—*ASS1*, *HDAC2*, *SIRT3*, and *VEGFA*—as Co-DEGs in patients with Alzheimer’s disease (AD) and OSA. Through GSEA, we identified significantly enriched signaling pathways related to inflammation, stress response, cell proliferation, and differentiation in OSA and NAFLD, with the TGF BETA SIGNALING PATHWAY being shared between the two diseases. Using PPI analysis, we found that besides *ASS1* and *HDAC2*, other Co-DEGs exhibited interacting relationships, indicating a synergistic impact on the diseases through physical interactions, co-expression, mutual prediction, co-localization, genetic interactions, and shared protein domain connections. Notably, *SIRT3* and *VEGFA* scored the highest in the MCC algorithm, highlighting their significance in the core of both diseases. By comparing the ROC curves, we identified key predictor genes for OSA and NAFLD. In the OSA group, *HDAC2* and *VEGFA* showed high accuracy (AUC > 0.7) in distinguishing case and normal groups, with significant differences in their expression levels (*p* < 0.05). Likewise, in the NAFLD group, *HDAC2*, *SIRT3*, and *VEGFA* were accurate predictors (AUC > 0.7) for case and normal groups, with differential expression observed for *ASS1* (*p* < 0.05), *HDAC2* (*p* < 0.01), and *SIRT3* and *VEGFA* (*p* < 0.001). The genes and pathways identified in this study are linked to oxidative stress, inflammatory responses, and disruptions in circadian rhythms, suggesting common pathways through which OSA and NAFLD may interact, aligning with previous research findings (Friedman et al., 2018).

OSA causes organ lesions based on chronic intermittent hypoxemia (CIH), which triggers a series of metabolic reactions. CIH aggravates the body's oxidative stress response by triggering relative oxygen production in OSA patients and leading to an oxidative/antioxidant imbalance (Hoffmann et al., 2013). At the same time, due to repeated obstruction events of OSA, IH circulation occurs, releasing hypoxia-inducible factors (HIF-1α) and inflammatory factors, causing mitochondrial dysfunction, and rapid reoxidation of transient hypoxic tissues may lead to tissue damage and release reactive oxygen species (ROS), which are key activators of inflammatory pathways (Bonsignore and Eckel, 2009), which can further induce oxidative stress and inflammatory response and trigger hepatocyte fat deposition and liver damage through multiple downstream mechanisms, leading to the occurrence or progression of NAFLD. Related studies have shown significant progression of NAFLD in patients with OSA and hypoxemia compared with young patients with non-OSA and hypoxemia, suggesting that CIH-mediated oxidative stress and inflammatory responses may be an important trigger for NAFLD progression (Sundaram et al., 2016). Studies based on animal experiments have shown that under the IH condition of mouse hepatocytes, the expression level of the HIF-1α protein is increased, but it is not expressed at a normal oxygen concentration (Mesarwi et al., 2016), which indicates that CIH can activate the liver to produce HIF-1α, causing oxidative stress damage in liver cells. In addition, patients with OSA have circadian rhythm disorders, such as sleep and wake time imbalances, eating rhythm disorders, and hormone circadian rhythm disorders. Circadian rhythms regulate the activity of various organs in the body, and their disturbances can lead to a variety of complications, including metabolic, cardiovascular, and neurodegenerative diseases (McHill and Wright, 2017; Lemmer and Oster, 2018; Uddin et al., 2021). The relationship between OSA and circadian rhythm disturbance may be bidirectional, with the endogenous circadian system regulating the pattern of respiratory OSA events and OSA modulating the circadian system through repeated upper airway obstruction, leading to sleep fragmentation and IH (Šmon et al., 2023). The induced circadian clock disruption may be a potential signaling pathway that is associated with the development and exacerbation of metabolic syndrome in patients with OSA (Malicki et al., 2022). Circadian rhythm disturbances are also closely related to NAFLD. The pathogenic mechanism of NAFLD development is complex, and it is mainly believed that metabolic disorders and genetic background are involved in this process; circadian rhythm disorders can participate in the development and progression of NAFLD by participating in the regulation of hormones and metabolic homeostasis and ultimately participate in the occurrence of HCC (Gnocchi et al., 2019). Relevant studies have proposed that restricting eating to a specified daily interval can synchronize the central and peripheral circadian rhythms so as to prevent or even treat metabolic syndrome and hepatic steatosis, indicating that circadian rhythms may be the target of NAFLD treatment (Saran et al., 2020). Many studies (Gabryelska et al., 2022; von Allmen et al., 2018) have highlighted the relationship between circadian rhythms and hypoxia, and oxygen acts as a circadian synchronizer that is essential for maintaining circadian balance *in vivo* (Hunyor and Cook, 2018). IH is one of the main pathophysiological features of OSA and its related comorbidities, which can induce circadian rhythm disorders between tissues (Hunyor and Cook, 2018). In summary, we hypothesize that OSA may play a role in the pathogenesis of NAFLD through IH-induced oxidative stress, chronic inflammatory response, and circadian rhythm disruption.

Assuming a causal relationship between *ASS1* and the inflammatory response, our investigation uncovered the potential of *ASS1* as a pivotal gene differentiating between OSA and NAFLD. Thus, we posit that chronic inflammation potentially mediates the interaction mechanism between OSA and NAFLD. Argininosuccinate synthetase 1 (ASS1), predominantly present in hepatic cells, serves as a crucial enzyme in arginine metabolism, intricately linked to urea and nitric oxide synthesis. Prior studies indicate the altered expression of various metabolites (Park et al., 2005), notably reduced arginine levels in liver injury scenarios (Sharma et al., 2012), signifying the downregulation of *ASS1* during hepatic damage. L-arginine, an indispensable amino acid, plays a fundamental role in diverse biosynthetic pathways and regulatory functions such as immune modulation, neural activity, and endothelial homeostasis. Furthermore, it functions as a precursor for protein, creatine, and polyamine synthesis, besides aiding in ammonia neutralization and liver detoxification in patients with hepatic conditions (Nanji et al., 2001). Animal models demonstrate the protective effects of arginine supplementation in liver injury syndromes, such as lipopolysaccharide-induced injury, hepatic ischemia–reperfusion injury, and acute cholestatic liver injury (Muriel and González, 1998; Taha et al., 2009; Li et al., 2012). The regulation of *ASS1* expression primarily hinges upon various stimuli like hormones, inflammatory mediators, cytokines, and lipopolysaccharides. The literature also links elevated inflammatory markers in OSA patients—such as C-reactive protein, tumor necrosis factor-alpha, interleukins 6 and 8, intercellular adhesion molecule, and vascular cell adhesion molecule—with the severity of OSA assessed by the apnea–hypopnea index (AHI) (Nadeem et al., 2013; Arnaud et al., 2020). We postulate that in the context of chronic intermittent hypoxia triggering inflammatory cascades, the ensuing hyperinflammation hampers *ASS1* expression, culminating in liver injury and NAFLD pathogenesis. Notably, *ASS1* exerts hepatoprotective effects in the interplay between OSA and NAFLD; hence, arginine supplementation holds promise as a therapeutic intervention for liver ailments.

*VEGFA* and *HDAC2* are currently believed to be closely linked to oxidative stress and play pivotal roles in the interplay between OSA and NAFLD. Oxidative stress responses leading to tissue ischemia and hypoxia, including ROS, vascular endothelial growth factor (VEGF), advanced glycosylation end products (AGEs), and plasminogen activator inhibitor-1 (PAI-1), have been identified in individuals with Obstructive sleep apnea hypopnea syndrome (OSAHS), potentially contributing to the onset and progression of vascular disease (Yamamoto et al., 2008; Assoumou et al., 2012). In the context of chronic liver disease, vascular endothelial growth factor A (VEGFA) acts as a crucial regulator of angiogenesis, contributing to endothelial dysfunction and immune cell infiltration (Bocca et al., 2015; Apte et al., 2019). Abnormal angiogenesis appears to be inherently associated with fibrosis throughout the course of chronic liver disease. Research indicates that VEGFA mediates hepatic stellate cell (HSC) activation, promoting the advancement of NAFLD-HCC. Pathological angiogenesis linked to VEGFA plays a significant role in the progression of NAFLD, fostering inflammation, fibrosis, and development of hepatocellular carcinoma (Shen et al., 2022). Notably, fibrosis stands out as the principal risk factor for malignant transformation in NAFLD patients (Adams et al., 2005; Powell et al., 2021), with HSC being a key target of VEGFA. In conclusion, it is posited that the oxidative stress response in OSA patients may enhance *VEGFA* expression, subsequently fueling NAFLD progression. Targeted pharmacological interventions involving *VEGFA* may serve to delay the advancement of chronic liver disease. Moreover, recent studies have associated *HDAC2* with the modulation of ROS levels. Histone deacetylase-2 (HDAC2), a member of the histone deacetylase family, participates in the regulation of chromatin structure during transcription. The levels of histone acetylation play a critical role in maintaining nuclear stability, gene expression, physiological function, and chromatin structure in hepatocytes (Cai et al., 2018; Ferriero et al., 2018). Dysregulation of histone deacetylation has been implicated in the initiation and advancement of liver disease (Cao et al., 2013). NASH represents the more severe stage of NAFLD, with the ongoing accumulation of ROS, oxidative stress, mitochondrial dysfunction, triglycerides, and lipid-toxic metabolites being key factors in NASH development (Fukushima et al., 2009). Inhibiting *HDAC2* expression could potentially aid in NASH prevention (Zhong et al., 2017). Studies have indicated a correlation between *HDAC2* and ROS (van de Ven et al., 2017), with elevated *HDAC2* expression potentially serving as a crucial link in the onset of OSA-induced NAFLD. Therefore, addressing the delicate balance between hepatic acetylation and ROS levels may offer a novel treatment avenue for individuals with OSA and NAFLD.

*SIRT3* is implicated in oxidative stress and disruptions of the circadian rhythm. Mitochondrial sirtuin 3 (SIRT3) is an NAD ±-dependent protein deacetylase crucial for the maintenance of redox balance and lipid homeostasis, thereby ensuring cellular equilibrium (Ahn et al., 2008). Its deficiency can result in hepatic steatosis. *SIRT3* modulates the activity of various proteins by deacetylating lysine residues, thus orchestrating mitochondrial biogenesis, energy production, and the equilibrium of ROS (van de Ven et al., 2017). Studies indicate that SIRT3 regulates ATP synthesis in the mitochondria by influencing the respiratory chain functioning (Ahn et al., 2008). Moreover, SIRT3 safeguards mitochondrial activity by controlling ROS production through multiple substrates, including superoxide dismutase 2 (SOD2) and forkhead box O3 (FOXO3A) (Qiu et al., 2010; Yu et al., 2012). Notably, experiments on animals lacking *SIRT3* and fed a high-fat diet reveal more severe hepatic steatosis compared to wild-type mice (Hirschey et al., 2011). Furthermore, recent research (Chang and Guarente, 2014) underscores the significant role of mitochondrial sirtuin (SIRT) in regulating human metabolism and circadian rhythms by influencing central and peripheral circadian clocks (Masri, 2015). Studies on mice deficient in mitochondrial sirtuin 1 (SIRT1) show circadian rhythm disturbances, indicating a link between SIRT and the circadian cycle (Wang et al., 2016). Interestingly, individuals with OSA exhibit reduced levels of serum SIRT1, which can be restored post-CPAP therapy (Chen et al., 2015). Consequently, it is hypothesized that akin to *SIRT1*, *SIRT3* might play a pivotal role in circadian rhythm disruptions in OSA patients, potentially triggering metabolic impairments and ultimately leading to NAFLD. Hence, the downregulation of *SIRT3* could be a critical factor in the development of NAFLD in OSA patients and a protective mechanism against the disease.

In this study, *HDAC2*, *SIRT3*, and *VEGFA* showed high accuracy in predicting both diseases, indicating their importance as key predictor genes for OSA and NAFLD. These genes likely do not act independently in the two diseases; rather, their interaction may play a significant role in the development and progression of OSA and NAFLD. We hypothesize that IH-induced oxidative stress, chronic inflammatory response, and disruption of circadian rhythm could be underlying mechanisms linking OSA to the development of NAFLD. Our findings offer promising target genes for future basic experimental and clinical studies, serving as potential biomarkers for diagnosing and treating OSA and NAFLD. Additionally, through GSEA, we identified signaling pathways related to inflammation, stress response, cell proliferation, and differentiation in OSA and NAFLD, suggesting avenues for further research into these diseases and their associated tumors. However, this study has limitations. It is a retrospective analysis based on existing databases and public information, limiting the ability to analyze differences and sensitivities of samples based on specific clinical characteristics. Causality cannot be determined, and the small sample size underscores the need for a future large-scale, multicenter, randomized controlled and prospective observational study to verify the identified genes and establish causal relationships between OSA and NAFLD. Furthermore, while key predictor genes shared by OSA and NAFLD have been identified, further research is needed to elucidate their molecular and biological functions in these diseases. Extensive basic and clinical investigations of these genes are essential for understanding their interaction mechanisms and utilizing them for clinical diagnosis, treatment, and disease prediction.

## Conclusion

This study is the first to utilize bioinformatics methods in investigating the shared predictor genes of OSA and NAFLD. The research identified four key genes that have predictive value for both conditions. These key genes offer novel insights into the potential mechanistic interactions between OSA and NAFLD, thereby guiding future basic research and clinical endeavors. Furthermore, the study presents potential diagnostic and therapeutic markers for individuals with OSA and NAFLD.

## Data Availability

The original contributions presented in the study are included in the article/Supplementary Materials, further inquiries can be directed to the corresponding authors.
