# Supplementary material for: An integrated bioinformatics analysis to identify the shared biomarkers in patients with obstructive sleep apnea syndrome and nonalcoholic fatty liver disease
Source: Front Genet. 2024 Jul 16;15:1356105. doi: 10.3389/fgene.2024.1356105 (PMC11286465; doi:10.3389/fgene.2024.1356105)
Supplement: Supplementary file 1 [file DataSheet1.PDF]

Supplementary Material

TABLE 1. List of dataset information.

items	GSE38792	GSE89632
Platform	GPL6244	GPL14951
Species	Homo sapiens	Homo sapiens
Disease	OSA	NAFLD
Tissue	Visceral adipose tissue	Liver
Samples in the Case Group	39	10
Samples in the Normal group	24	8
Reference	(3)	(4)

OSA: obstructive sleep apnea; NAFLD: nonalcohol fatty liver disease.

TABLE 2.GSEA analysis of dataset GSE38792.

Description	setSize	enrichment score	NES	p-value	q-value
REACTOME CELL CYCLE MITOTIC	453	0.399311539	1.955092057	0.000139218	0.007625044

WP VEGFAVEGFR2 SIGNALING PATHWAY*	395	0.318495785	1.542000725	0.000141143	0.007625044
REACTOME CLASS I MHC MEDIATED ANTIGEN PROCESSING PRESENTATION	333	0.35495257	1.694858866	0.00014374	0.007625044
REACTOME M PHASE	316	0.43204613	2.051897554	0.000145033	0.007625044
REACTOME ASPARAGINE N LINKED GLYCOSYLATION	266	0.387808776	1.811180513	0.000147689	0.007625044
REACTOME ORGANELLE BIOGENESIS AND MAINTENANCE	250	0.387058632	1.795034965	0.000148368	0.007625044
REACTOME PLATELET ACTIVATION SIGNALING AND AGGREGATION	244	0.345733742	1.59952342	0.00014839	0.007625044
REACTOME DNA REPAIR	251	0.391426592	1.814982473	0.000148633	0.007625044
REACTOME CELL CYCLE CHECKPOINTS	233	0.435028556	2.001351684	0.000149723	0.007625044
REACTOME RHO GTPASE EFFECTORS	238	0.379048484	1.746110214	0.000150083	0.007625044

REACTOME DEUBIQUITINATION	220	0.360639403	1.647550585	0.000150784	0.007625044
REACTOME FCERI-MEDIATED NF KB ACTIVATION	77	0.404494579	1.585842553	0.006012024	0.064962718
REACTOME P130CAS LINKAGE TO MAPK SIGNALING FOR INTEGRINS	15	0.638084798	1.733972301	0.007993909	0.076478233
WP TGFBETA SIGNALING PATHWAY*	128	0.344302402	1.46128931	0.013130504	0.09869798
REACTOME SIGNALING BY HEDGEHOG	137	0.335039805	1.435574434	0.013992686	0.101217951

GSEA: Gene Set Enrichment Analysis.* Represents the signaling pathways shared by both diseases.

TABLE 3. GSEA analysis of dataset GSE89632.

Description	set Size	enrichment score	NES	p-value	q-value
REACTOME GPCR LIGAND BINDING	435	-0.345270488	-1.582097984	0.000133476	0.005819216
REACTOME SIGNALING BY INTERLEUKINS	427	-0.486109881	-2.223201608	0.000134336	0.005819216

WP VEGFAVEGFR2 SIGNALING PATHWAY*	411	-0.360100378	-1.641413424	0.000135135	0.005819216
KEGG OLFACTORY TRANSDUCTION	381	-0.617695066	-2.797025693	0.000136705	0.005819216
REACTOME OLFACTORY SIGNALING PATHWAY	374	-0.607846828	-2.745263814	0.000137438	0.005819216
NABA SECRETED FACTORS	325	-0.443081706	-1.977235319	0.000139276	0.005819216
REACTOME CLASS A 1 RHODOPSIN-LIKE RECEPTORS	307	-0.381423481	-1.692225661	0.000141423	0.005819216
WP NUCLEAR RECEPTORS META PATHWAY	307	-0.424678126	-1.884129471	0.000141423	0.005819216
REACTOME EXTRACELLULAR MATRIX ORGANIZATION	286	-0.428167739	-1.886043407	0.000142531	0.005819216
WP IL18 SIGNALING PATHWAY	262	-0.463689704	-2.028176355	0.000143719	0.005819216
NABA CORE MICROSOME	255	-0.407800593	-1.77711959	0.000144823	0.005819216

KEGG JAK-STAT SIGNALING PATHWAY	151	-0.534655611	-2.187984929	0.000153468	0.005819216
WP TGFBETA SIGNALING PATHWAY*	129	-0.537025337	-2.149688837	0.000157109	0.005819216
WP PI3KAKT SIGNALING PATHWAY	333	-0.362479724	-1.620986548	0.000139005	0.005819216
KEGG MAPK SIGNALING PATHWAY	257	-0.423841351	-1.849335982	0.000144363	0.005819216

GSEA: Gene Set Enrichment Analysis.* Represents the signaling pathways shared by both diseases.

TABLE S1. List of gene symbols of UPRmtRGs.

Gene symbol					
ABCB10	AFG3L2	ALKBH1	APP	ASNS	ASS1
ATF3	ATF4	ATF5	BATF	BATF2	BATF3
CAT	CEBPA	CEBPB	CEBPD	CEBPE	CEBPG
CHCHD2	CLK1	CLPP	CLPX	COQ7	COX8A
CREB3	CREBZF	DDIT3	DEFA5	EIF2AK1	EIF2AK2
EIF2AK3	EIF2AK4	EIF2B1	EIF2S1	ENDOG	ESR1

FAHD1	FGF21	FIS1	FOS	FOSL1	FOXO3
GDF15	HDAC1	HDAC2	HSF1	HSP90AA1	HSPA14
HSPA9	HSPD1	HSPE1	HTRA2	HTT	IL20RB
IMMP1L	IMMP2L	JUN	JUNB	KIAA0391	LONP1
LRPPRC	MAF	MAF1	MAFB	MAP3K4	MAPK14
MARS2	MIR382	MYC	NFE2	NFE2L1	NFE2L2
NFE2L3	NRF1	OMA1	OPA1	PARP2	PCK2
PDF	PHB1	PINK1	PITRM1	PMPCA	PMPCB
PPIF	PRKN	PRORP	SIRT1	SIRT3	SIRT7
SLC1A4	SLC1A5	SLC6A6	SOD2	SPG7	TARDBP
TFAM	TP53	TRAP1	TXN2	UBL5	VEGFA

UPRmtRGs: mitochondrial unfolded protein response-related genes.

TABLE S2. Co-DEGs in the PPI network ranked by MCC method.

Rank	Name	Score
1	VEGFA	4
1	SIRT3	4

3	ASS1	2
3	HDAC2	2

Co-DEGs: common differential expression genes. PPI: protein-protein interaction. MCC: maximal clique centrality.
